# Conference Report: INTERNATIONAL WORKSHOP ON INSTRUMENTED INDENTATION San Diego, CA April 22–23, 1995

**DOI:** 10.6028/jres.101.010

**Published:** 1996

**Authors:** Douglas T. Smith

**Affiliations:** Ceramics Division, National Institute of Standards and Technology, Gaithersburg, MD 20899-0001

## 1. Introduction

An international workshop was held on April 22–23, 1995, at the Town and Country Hotel in San Diego, to discuss the scientific and standardization issues associated with instrumented indentation, also known as dynamic hardness testing or depth-sensing, ultra-low-load, or nano indentation. The workshop was sponsored jointly by the NIST Standard Reference Materials Program (SRMP) and the Institute for Mechanics and Materials (IMM) in San Diego, with additional support for student travel from Nano Instruments, Inc., Oak Ridge, TN, and Instron Corporation, Canton, MA, and was part of the program of the 1995 International Conference on Metallurgical Coatings and Thin Films (ICMCTF95). The 96 attendees represented 14 U.S. and 6 foreign companies, 18 U.S. and 7 foreign universities, and 15 national laboratories, including NIST, the National Physical Laboratory (NPL) in England and the Commonwealth Scientific and Industrial Research Organization (CSIRO) in Australia. The 2-day program consisted of 22 oral presentations, 22 poster presentations, and several open discussion sessions. This report summarizes the workshop program. More detailed proceedings of the workshop will be available as a NIST Special Publication.

## 2. Background

Indentation has been used for many years to measure the hardness of materials. The technique involves pushing an indenter tip, typically a sphere, cone or diamond pyramid, into a material under controlled load, then measuring the size of the residual impression. This testing is economical, both in terms of equipment and time, and produces a reliable hardness measurement in macroscopic specimens.

Recently, however, a more sophisticated form of indentation testing, known as “instrumented” or “depth sensing” indentation, has been developed that offers significant advantages over traditional indentation. In an instrumented indentation system, an indenter tip is loaded onto a specimen under computer control of either the load or the displacement or both, and load, displacement and time are recorded continuously throughout the loading-unloading cycle. These data form what is known as a “load-displacement curve,” and they contain a wealth of information about the elastic, plastic and time-dependent deformation behavior of the material being tested. Imaging of the residual impression is not necessary, although scanning and transmission electron microscopy and atomic force microscopy can yield useful information about deformation mechanisms. The technique is routinely used to determine mechanical properties from indentations that are sub-micrometer in size, and is considered to be of particular value in evaluating the mechanical properties of thin films on substrates. When used to make sub-micrometer-scale indentations, the technique is often referred to as ultra-low-load or nano indentation. Several national standards laboratories, including NIST, NPL, and CSIRO, have built their own instrumented indenters, and at least four companies now market commercial machines with varying levels of performance and sophistication. Instrumented indenters are currently used in a number of industrial research laboratories, including 3M, Intel, Rockwell, Kodak, United Technologies, and several IBM sites.

Despite the ability of instrumented indentation to make micrometer- and nanometer-scale mechanical properties measurements, use and acceptance of the technique is hampered by a lack of standardization in the field. Not only are there no standard reference materials for use with instrumented indenters, there are differences of opinion about exactly how best to analyze load-displacement curves to yield quantities such as hardness and Young’s modulus. The purpose of the workshop was to bring together researchers from universities and from standards and other government laboratories with industrial users of the technique to outline areas of agreement and disagreement, and to begin the process of establishing standard, or at least recommended, test methods and data analysis techniques, and, ultimately, standard reference materials. The specific goals of the workshop were:

- to assess the current industry use of instrumented indentation, and to hear from industry users about their needs for standardization in the field;
- to provide attendees, particularly those from industry, with detailed, up-to-date information on what material properties are currently being measured with the technique, and how those properties can be extracted from load-displacement curves;
- to discuss the “state of the art” in analytical and finite-element modeling of the indentation process; and
- to discuss standardization issues in instrumented indentation, including a) current standardization efforts within international standards committees, b) machine and tip shape calibration techniques, d) the effects of test parameters (loading rates, dwell times, etc.) on results, and d) possible candidates for standard reference materials.

## 3. Workshop Program

The workshop ran for 2 full days, and included four sessions of oral presentations and discussion plus an evening poster session. Listings of the oral and poster presentations are in Appendices A and B, respectively. A brief summary of each session follows.

### 3.1 Industrial Applications

Discussion Leader: Trevor Page, University of Newcastle. The workshop began Saturday morning with a set of four talks from industrial users of instrumented indentation, to hear from them what materials characterization is currently being done in industry with the technique, and what problems they might be having applying the technique. Richard White, from IBM’s Storage Systems Division, San Jose, began by describing how he is using instrumented indentation to characterize the mechanical properties of the various hard materials used in layered magnetic hard disk drive systems, and how those properties correlate with wear performance. Kevin O’Connor, from Eastman Kodak, Rochester, then described the problems of studying much softer coatings, such as the polymers and gelatins used in photographic materials. These materials often exhibit strong creep and stress relaxation effects. Dr. O’Connor suggested that additional work in the analysis of highly dissipative, viscoelastic systems, particularly layered systems, would be a great help to him in interpreting his indentation data. Harry Fujimoto presented work being done at Intel Corporation on the use of indentation to induce delamination in layered systems as a means of measure interfacial adhesion. Results on several systems, including a tungsten coating on a softer metal substrate, were discussed. Finally, Clark Cooper, from United Technologies Research Center (UTRC), summarized his experience with instrumented indentation. His comments were of particular interest because UTRC purchased one of the first commercial indenters from Nano Instruments, Inc., and has logged 10 years of use with it.

### 3.2 Determining Material Properties

Discussion Leader: William Nix, Stanford University. The purpose of the second session, “Determining Material Properties,” was to present a series of talks from an experimental perspective that described which material properties can, in principle, be determined using instrumented indentation, and how they should be determined. The session actually began before lunch, with two overview talks to set the tone for the afternoon. First Warren Oliver, from Nano Instruments, Inc., gave a brief overview of the use of sharp diamond indenters. He described in general terms the elastic and plastic response of materials to sharp indenters, methods of calculating hardness and Young’s modulus, and problems inherent in determining the tip shape and contact area between tip and sample. His talk was followed by one from Michael Swain, CSIRO. Dr. Swain focused primarily on the use of spherical indenters, and their advantages over sharp indenters for determining the full stress-strain response of materials. He also described errors that can result if the indenter tip shape is not perfectly spherical, and suggested a procedure to correct for this in the analysis.

Six talks were presented in the afternoon, each presenting a detailed discussion of an experimental method for determining a specific material property, or set of properties. David Rowcliffe, from the Royal Institute of Technology (KTH) in Stockholm, outlined a method of analyzing Vickers load-displacement curves, supported by three-dimensional finite element analysis, to determine hardness, yielding stress, strain hardening and plastic zone size.

Two talks then followed that dealt specifically with coated systems. First, Trevor Page, from the University of Newcastle, gave a detailed accounting of problems associated with the use of instrumented indentation to characterized coating/substrate systems, and stressed that great care must be taken to accurately interpret results from these systems. Neville Moody, of Sandia National Laboratories, described how his group had combined nanoindentation, continuous scratch testing, and high resolution transmission electron microscopy to study tantalum nitride films and their interface to sapphire.

After a short break, William Gerberich presented work by his group at the University of Minnesota on the yield of materials at very low loads. He showed evidence for initial yield in Fe with mass fraction of Si, w(Si), of 0.03 at a load of 150 μN under a Berkovich diamond indenter.

The final two talks in the session dealt with the determination of time-dependent properties. Jean-Luc Loubet, of the Centre National de la Recherche Scientifique (CNRS, France), described his current work using an oscillating-load technique to study viscoelastic behavior. Howard Poisl, from the University of Arizona, showed, using experiments on amorphous selenium, how indentation creep and indentation strain rate measurements could be related to more conventional creep measurements.

### 3.3 Poster Session

A poster session, containing 22 entries, was held on Saturday evening. The posters covered a broad range of experimental and theoretical topics, from standardization, calibration and instrumentation issues to analytical analyses of contact stresses in layered systems. The session was well-attended and generated a great deal of animated discussion. A list of poster presentations is given in Appendix B.

### 3.4 Modeling of the Indentation Process

Discussion Leader: John Pethica, Univ. of Oxford. The Sunday morning session consisted of four talks on theoretical and modeling aspects of instrumented indentation. It began with a talk by George Pharr, of Rice University, comparing experimental and finite element simulation results on the effect of residual stress in a material on its measured hardness and modulus. Although the experimental results indicated a dependence of both properties on residual stress, the finite element simulation indicated that the effect was not real, but was instead a result of the residual stress changing the amount of pile-up around the contact site, thus causing the actual contact area to differ significantly from the calculated area.

Joost Vlassak then described his work at Stanford University on the effects of elastic anisotropy on the measured “indentation modulus,” which is not in general the Young’s modulus in the direction of indentation for anisotropic materials. In the next talk, Antonios Giannakopoulos presented results of three-dimensional finite element modeling of the sharp indentation process, with and without friction at the contact, and with different degrees of strain hardening.

Finally, Subbiah Ramalingam, from the University of Minnesota, presented an analytical approach to the calculation of elastic stresses that develop in film/substrate systems. The approach permitted an analysis of the stress fields within each material and at the interface between film and substrate.

### 3.5 Methodology and Standardization

Discussion Leader: Douglas Smith, NIST. The final session combined several aspects of instrumentation and standardization. John Pethica, from the University of Oxford, opened the session with some cautionary remarks about problems that are encountered when performing indentation experiments on length scales approaching atomic dimensions. He cited meniscus and surface adhesion forces as having significant effects on measurements in that regime.

The next two talks dealt with the use of atomic force microscopy (AFM) technology for mechanical property measurement. Steve Hues presented work from NRL on the use of AFMs to make quantitative modulus measurements, by replacing piezoelectric actuators with electrostrictive materials in an effort to eliminate hysteresis and creep, and by using small glass spheres as indenters, for their more easily characterized tip geometry. Jack Houston, from Sandia, then described a novel force-balanced AFM tip support, developed at Sandia, that is capable of accurately recording attractive tip-substrate interactions through a feedback system that gives the tip support a near-infinite effective stiffness. Warren Oliver returned to finish the instrumentation part of the session with a discussion of techniques for calibrating the shape of sharp indenter tips.

The session then concluded with two talks directly addressing standardization efforts in the instrumented indentation community. First, Stuart Saunders, from NPL, presented the results of research, partly funded by the European Commission, on the “Measurement of Hardness (Mechanical Properties) of Surfaces.” The work involved developing machine and tip calibration procedures, as well as conducting round-robin testing with three commercial machines at 14 sites. Hans-Hermann Behncke, from Helmut Fischer Company, described work being done in Germany towards a draft ISO standard (TC 164, WG 3) for a quantity referred to as “Universal Hardness,” based on Vickers indentation at depths greater than 3 μm. Dr. Behncke also presented results from round robin testing of samples consisting of titanium nitride coatings on steel.

## 4. Summary

The workshop concluded with an open discussion period at the end of the Sunday afternoon session. Although many topics were addressed, the theme throughout the discussion was to determine to what extent “standardization” of instrumented indentation, in any sense of the word, was desirable, or even possible in the near future. Just the array of names for the technique itself highlights the lack of consensus in the field; it is referred to as *instrumented indentation, dynamic hardness testing, depth-sensing indentation, continuously recording indentation technique*, and more. At very low loads, the terms *nanoindentation, ultra-micro-indentation* and *ultra-low-load indentation* are all used. The views of the participants on issues like standard test procedures and standard reference materials ranged from considering them to be essential and long overdue to considering such efforts premature and essentially impossible to implement until the indentation process is better understood. One of the more memorable comments in the discussion came from Bill Nix, when he stated that the many different approaches to the problems presented in the workshop represented a healthy level of scientific activity, and that people should not be overly concerned about the lack of consensus. His remarks were greeted with applause.

There was however one area where many participants agreed. It was noted that very often when values for hardness, modulus or other quantities obtained by instrumented indentation are reported in the literature, little information is given on the test parameters that were used in the measurements. It would be easier for people to compare their results to those by others in the literature if authors gave, and editors required, a minimum amount of information on technique from those publishing indentation results. That information should include experimental parameters like the loads used, the depths of the indents at those loads, loading rates, and calibration corrections applied, as well as the precise definition of the result (e.g., hardness, modulus) and how it was obtained from the load-displacement curves. If this information is available, people will at least be able to determine whether they can directly compare their results to those from another group.

There was also discussion, at several points during the workshop, about what materials might make good standard reference materials. An ideal material should be easy to polish reproducibly, as surface finish becomes critical at low loads, and should be as elastically isotropic as possible, so that the precise orientation between sample and indenter geometry is not critical. Single crystal tungsten and several glasses, including pure fused silica and BK-7 glass, have been used in round-robin testing and are possible candidates.

By all comments received by the Chairman during and following the workshop, the participants felt that the workshop had been a very valuable meeting, despite the failure of the group to adopt any firm recommendations for test or analysis procedures. It brought together many of the most experienced people in the field to exchange ideas, and many participants expressed a desire to meet on a more regular basis. Several sessions at the 1996 ICMCTF meeting will include talks on instrumented indentation.

## Appendix A. Oral Presentations

**Douglas T. Smith**, NIST, Gaithersburg, MD Opening Remarks

1. **Richard L. White**, IBM, San Jose, CA
  “Nanoindentation of Magnetic Storage Materials”
2. **Kevin O’Connor**, Eastman Kodak Company, Rochester, NY
  “The Use of Instrumented Indentation in the Design of Multilayer Coating Structures for Imaging Applications”
3. **Harry H. Fujimoto**, Intel Corporation, Santa Clara, CA
  “Microindentation for Electronic Thin Films”
4. **Clark V. Cooper**, United Technologies Research Center, East Hartford, CT
  “The Use of a Nanoindenter I: Case Histories and Lessons Learned”
5. **Warren C. Oliver**, Nano Instruments, Inc., Oak Ridge, TN
  “Measuring Hardness, Modulus, Creep and Fracture Toughness Using Instrumented Indentation Tests”
6. **Michael V. Swain**, CSIRO, Lindfield, NSW, Australia
  “Simple Procedures for Obtaining and Analysing Force Displacement Data with Small, Spherical-Tipped Indenters”
7. **David J. Rowcliffe**, Royal Institute of Technology, Stockholm, Sweden
  “Controlled Indentation: A General Approach to Determine Mechanical Properties of Materials”
8. **Trevor F. Page**, Univ. of Newcastle, Newcastle upon Tyne, U.K.
  “Procedures for the Nanoindentation Testing of Coated Systems”
9. **Neville R. Moody**, Sandia National Laboratories, Livermore, CA
  “Thin Film Property Measurements Using Nanoindentation and Microscratch Techniques”
10. **William W. Gerberich**, Univ. of Minnesota, Minneapolis, MN
  “Multiple Yield Phenomena under Light Contacts”
11. **Jean-Luc Loubet**, Centre National de la Recherche Scientifique, Cedex, France
  “Some Measurements of Creep and Viscoelastic Behavior with the Help of Indentation Tests”
12. **W. Howard Poisl**, Univ. of Arizona, Tucson, AZ
  “Determination of the Relationship between Indentation and Uniaxial Creep Strain Rates in Amorphous Selenium”
13. **George M. Pharr**, Rice Univ., Houston, TX
  “Effects of Residual Stress on the Measurement of Mechanical Properties using Instrumented Indentation”
14. **Joost J. Vlassak**, Stanford Univ., Stanford, CA
  “Measuring the Indentation Modulus of Elastically Anisotropic Solids”
15. **Antonios E. Giannakopoulos**, Royal Institute of Technology, Stockholm, Sweden
  “3-D Finite Element Modeling of Sharp Indentations”
16. **Subbiah Ramalingam**, Univ. of Minnesota, Minneapolis, MN
  “Analytical Modeling of Stresses in the Film/Substrate System, and their Significance for Indentation Testing”
17. **John B. Pethica**, Univ. of Oxford, Oxford, U.K.
  “Detecting the First Contact—The Role of Adsorbed Molecules”
18. **Steven M. Hues**, Naval Research Laboratory, Washington, DC
  “Nanoindentation Using the AFM: Considerations for the Quantitative Measurement of Mechanical Properties”
19. **Jack E. Houston**, Sandia National Laboratories, Albuquerque, NM
  “Interfacial Force Microscopy Measurements of the Nanomechanical Properties of Materials”
20. **Warren C. Oliver**, Nano Instruments, Inc., Oak Ridge, TN
  “Tip Geometry Calibration: Is Fused Silica an Effective Standard for Ultra-Low Load Indentation?”
21. **Stuart R. J. Saunders**, National Physical Laboratory, Teddington, Middlesex, U.K.
  “Calibration and Validation of Depth-Sensing Indentation: The European Dimension”
22. **Hans-Hermann Behncke**, Helmut Fischer Co., Germany
  “European Efforts under ISO TC164 for a Dynamic Loading Hardness Test Specification”

## Appendix B. Poster Presentations

1. **W. R. Newson and S. Saimoto**, Queen’s University, Kingston, Ontario, Canada
  “Determination of Thermodynamic Response of Polymers by Micro-Indentation Rate Changes”
2. **L. Riester and M. K. Ferber**, Oak Ridge National Laboratory, Oak Ridge, TN
  “Artifacts in Nanoindentation Procedures”
3. **Th. Dietz, A. Schultz, H. Vetters, and P. Mayr**, Institut für Werkstofftechnik, Bremen, Germany
  “Development of Standards for the Validation of Hard, Thin Coatings”
4. **W. J. Meng and G. L. Eesley**, General Motors, Warren, MI
  “Growth and Mechanical Anisotropy of TiN Thin Films”
5. **M. P. de Boer and W. W. Gerberich**, University of Minnesota, Minneapolis, MN
  “Adhesion of Metal Interconnects by Microwedge Indentation”
6. **K. B. Yoder, M. F. Tambwe, and D. S. Stone**, University of Wisconsin, Madison, WI
  “Assortment of Techniques in Instrumented Indentation”
7. **E. T. Lilleodden, W. Bonin, J. Nelson, J. T. Wyrobek, and W. W. Gerberich**, Univ. of Minnesota, and Hysitron, Inc., Minneapolis, MN
  “In Situ Imaging of Ultra-Light-Load Indents into GaAs and Fe-3wt% Si”
8. **D. Zirkin, B. Farber, and A. Heuer**, Case Western Reserve University, Cleveland, OH
  “Modification of the Nikon QM High Temperature Microhardness Tester to Obtain Load-Deflection Curves”
9. **B. Taljat, F. M. Haggag, and T. Zacharia**, Askerceva, Slovenia; Advanced Technology Corp., Oak Ridge, TN; and Oak Ridge National Laboratory, Oak Ridge, TN
  “New Analytical Procedure to Determine Stress-Strain Curve and Elastic Modulus from Instrumented Ball Indentation”
10. **S. Joshi and K. A. Richardson**, Univ. of Central Florida, Orlando, FL
  “Dynamic Hardness Testing of Infrared Materials”
11. **Kangjie Li and J. C. M. Li**, University of Rochester, Rochester, NY
  “Effect of Indenter Shape, Yield Stress and Modulus on the Indentation Process”
12. **F. Yang and J. C. M. Li**, University of Rochester, Rochester, NY
  “Viscosity Measurement by Impression Test”
13. **L. Zheng and S. Ramalingam**, University of Minnesota, Minneapolis, MN
  “Contact Stress Analysis of a Layered Solid Under a Rigid Indenter”
14. **K. R. Upadhyaya, S. Saimoto, and R. S. Timsit**, AMP of Canada, Ltd., Markham, Ontario, Canada; Queen’s University, Kingston, Ontario, Canada
  “Measurement of Strain-Rate Sensitivity by Nanoindentation Techniques”
15. **S. V. Hainsworth, H. Sjöström, J.-E. Sundgren, H. W. Chandler, and T. F. Page**, Univ. of Newcastle, Newcastle upon Tyne, U.K.; Linköping University, Linköping, Sweden
  “Analysis of Nanoindentation Load-Displacement Loading Curves”
16. **J. Marschall and F. Milstein**, Univ. of California, Santa Barbara, CA
  “Measurement and Theory of the Orientation Dependence of Knoop Microhardness in a Layered Single Crystal”
17. **K. E. Parmenter and F. Milstein**, Univ. of California, Santa Barbara, CA
  “Indentation and Strength of Fiber-Reinforced Aerogels”
18. **Y. Abd Al-Jahil and P. Nelson**, Univ. of Texas, Austin, TX
  “Study of Rock Indentation Fracture in Mechanical Excavation”
19. **A. S. Chekanov, T. S. Low, S. Alli, and B. Liu**, National Univ. of Singapore, Singapore
  “Indentation Testing of the Magnetic Thin-Film Head Alumina Substrate”
20. **A. Kant, R. O. Ritchie, M. D. Drory, R. H. Dauskardt, and I. G. Brown**, Lawrence Berkeley Laboratory, Berkeley, CA; Crystallume, Santa Clara, CA; and Stanford Univ., Stanford, CA
  “Bulk and Interfacial Crack-Propagation Behavior in CVD Diamond”
21. **K. Zeng, D. J. Rowcliffe, and P. Meier**, Royal Institute of Technology, Stockholm, Sweden
  “Residual Stress Fields at Indentations”
22. **O. Warren, P. R. Norton, W. K. Wan, J. E. Houston, C. A. DiRubio, and T. A. Michalske**, University of Western Ontario, London, Ontario, Canada; and Sandia National Laboratories, Albuquerque, NM
  “Interfacial Force Microscopy of the Nanomechanical Properties of Thin Films Formed on Carbon Steel from Zinc Dialkyl Dithiophosphate Antiwear/Extreme Pressure Oil Additives”

